# Supplementary material for: Performance of Winter Wheat Cultivars Grown Organically and Conventionally with Focus on Fusarium Head Blight and Fusarium Trichothecene Toxins
Source: Microorganisms. 2019 Oct 11;7(10):439. doi: 10.3390/microorganisms7100439 (PMC6843174; doi:10.3390/microorganisms7100439)
Supplement: Supplementary file 1 [file microorganisms-07-00439-s001.zip › Table S1.docx]

**Table S1.** Mean concentration (mg kg^-1^) of mineral compounds in soil samples of conventional and organic experimental fields

| **Mineral compound** | **Conventional** | **Organic** |
| --- | --- | --- |
| Ca | 3598 a | 4822 b |
| Na | 1178 a | 1378 b |
| K | 5610 b | 3881 a |
| Mg | 1883 b | 1641 a |
| Si | 323 a | 411 b |
| B | 285 a* | 311 b* |
| Cd | 0.203 b* | 0.072 a* |
| Co | 2.563 a | 2.397 a |
| Cr | 9.520 b | 7.907 a |
| Cu | 8.363 b | 6.578 a |
| Mn | 130 a | 147 b |
| Ni | 6.340 b* | 5.703 a* |
| Zn | 364 b | 52 a |

Values within the same row followed by the different letters are significantly different at the level of probability < 0.001 or * <0.05
