# Supplementary material for: Performance of Winter Wheat Cultivars Grown Organically and Conventionally with Focus on Fusarium Head Blight and Fusarium Trichothecene Toxins
Source: Microorganisms. 2019 Oct 11;7(10):439. doi: 10.3390/microorganisms7100439 (PMC6843174; doi:10.3390/microorganisms7100439)
Supplement: Supplementary file 1 [file microorganisms-07-00439-s001.zip › Table S2.docx]

**Table S2.** Phenotypic characters of 30 winter wheat cultivars grown in conventional and organic fields

| **No.** | **Cultivar** | **Conventional** | | | | **Organic** | | | |
| --- | --- | --- | --- | --- | --- | --- | --- | --- | --- |
|  |  | **Head-ing*** | **Flower-ing*** | **Plant height [cm]** | **Grain yield [kg]** | **Head-ing*** | **Flower-ing*** | **Plant height [cm]** | **Grain yield [kg]** |
| 1 | Akteur | 29 | 33 | 105.7 | 4.5 | 31 | 33 | 114.3 | 4.7 |
| 2 | Alcazar | 28 | 34 | 76.3 | 4.2 | 27 | 31 | 83.0 | 4.0 |
| 3 | Anthus | 26 | 28 | 95.3 | 5.5 | 25 | 28 | 104.7 | 5.1 |
| 4 | Batuta | 28 | 30 | 92.3 | 6.1 | 26 | 28 | 101.0 | 6.1 |
| 5 | Belenus | 29 | 30 | 83.7 | 6.3 | 29 | 30 | 101.3 | 5.1 |
| 6 | Bogatka | 27 | 29 | 85.0 | 4.2 | 27 | 29 | 103.0 | 5.4 |
| 7 | Boomer | 30 | 35 | 91.0 | 6.6 | 29 | 32 | 95.0 | 6.1 |
| 8 | Dorota | 30 | 30 | 88.7 | 5.6 | 27 | 29 | 87.0 | 4.6 |
| 9 | Figura | 25 | 28 | 96.0 | 3.6 | 25 | 27 | 109.0 | 5.7 |
| 10 | Garantus | 32 | 34 | 88.7 | 5.3 | 30 | 33 | 92.0 | 5.4 |
| 11 | Jenga | 32 | 34 | 82.3 | 6.5 | 28 | 29 | 89.0 | 6.7 |
| 12 | Kampana | 32 | 34 | 76.7 | 5.1 | 28 | 29 | 77.7 | 5.4 |
| 13 | Kohelia | 30 | 32 | 109.0 | 4.7 | 28 | 29 | 114.0 | 5.3 |
| 14 | Legenda | 30 | 32 | 117.0 | 3.8 | 27 | 29 | 107.3 | 5.1 |
| 15 | Ludwig | 27 | 30 | 118.7 | 4.6 | 25 | 26 | 108.3 | 5.9 |
| 16 | Markiza | 31 | 33 | 113.0 | 3.9 | 28 | 30 | 105.7 | 4.6 |
| 17 | Meteor | 31 | 34 | 103.3 | 5.6 | 28 | 30 | 105.0 | 5.6 |
| 18 | Mewa | 30 | 30 | 106.0 | 4.4 | 26 | 27 | 93.0 | 4.3 |
| 19 | Mulan | 28 | 30 | 97.0 | 6.0 | 26 | 27 | 98.0 | 6.0 |
| 20 | Muszelka | 30 | 32 | 81.7 | 4.8 | 27 | 30 | 73.7 | 5.2 |
| 21 | Naridana | 27 | 29 | 93.7 | 4.9 | 25 | 27 | 96.7 | 4.3 |
| 22 | Nateja | 30 | 31 | 116.7 | 2.9 | 26 | 27 | 106.3 | 3.1 |
| 23 | Ostka Strzelecka | 30 | 30 | 104.0 | 5.0 | 29 | 30 | 102.7 | 5.1 |
| 24 | Ostroga | 33 | 34 | 102.3 | 5.2 | 32 | 34 | 100.7 | 6.2 |
| 25 | Slade | 33 | 34 | 85.3 | 4.1 | 32 | 34 | 82.3 | 4.8 |
| 26 | Smuga | 24 | 26 | 105.3 | 5.9 | 24 | 26 | 105.0 | 5.4 |
| 27 | Sukces | 35 | 35 | 103.0 | 5.3 | 33 | 35 | 100.0 | 4.7 |
| 28 | Tonacja | 32 | 33 | 109.0 | 5.7 | 31 | 33 | 98.3 | 4.3 |
| 29 | Türkis | 30 | 31 | 95.0 | 5.5 | 29 | 30 | 102.3 | 4.2 |
| 30 | Zyta | 33 | 34 | 111.3 | 4.5 | 31 | 33 | 112.3 | 4.3 |
|  | Mean | 29.7 | 31.6 | 97.8 | 5.0 | 28.0 | 29.8 | 99.0 | 5.1 |

* - days from May 1
