# Supplementary material for: Performance of Winter Wheat Cultivars Grown Organically and Conventionally with Focus on Fusarium Head Blight and Fusarium Trichothecene Toxins
Source: Microorganisms. 2019 Oct 11;7(10):439. doi: 10.3390/microorganisms7100439 (PMC6843174; doi:10.3390/microorganisms7100439)
Supplement: Supplementary file 1 [file microorganisms-07-00439-s001.zip › Table S3.docx]

**Table S3.** Disease infection of 30 winter wheat cultivars grown in conventional and organic fields

| **No.** | **Cultivar** | **Conventional** | | | | | | **Organic** | | | | | |
| --- | --- | --- | --- | --- | --- | --- | --- | --- | --- | --- | --- | --- | --- |
|  |  | **FHBi (%)** | **LR (%)** | **ST B (%)** | **TS (%)** | **SNB (%)** | **YR (%)** | **FHBi (%)** | **LR (%)** | **ST B (%)** | **TS (%)** | **SNB (%)** | **YR (%)** |
| 1 | Akteur | 0.2 | 0 | 5 | 0 | 0 | 21.0 | 0.5 | 0 | 5 | 0 | 5 | 13.0 |
| 2 | Alcazar | 1.0 | 0 | 1 | 0 | 0 | 0 | 1.0 | 0 | 0 | 10 | 5 | 0 |
| 3 | Anthus | 0.4 | 5 | 5 | 0 | 0 | 0 | 0.3 | 1 | 1 | 5 | 5 | 0 |
| 4 | Batuta | 0.2 | 0 | 1 | 0 | 0 | 0 | 0.1 | 0 | 5 | 5 | 5 | 0 |
| 5 | Belenus | 1.7 | 20 | 5 | 0 | 0 | 0 | 1.8 | 10 | 0 | 10 | 5 | 0 |
| 6 | Bogatka | 0.5 | 0 | 5 | 0 | 0 | 13.2 | 0.4 | 0 | 5 | 0 | 5 | 0 |
| 7 | Boomer | 0.2 | 0 | 1 | 0 | 0 | 0 | 0.3 | 1 | 0 | 1 | 1 | 0 |
| 8 | Dorota | 0.1 | 1 | 5 | 0 | 0 | 0 | 0.1 | 1 | 5 | 0 | 1 | 4.0 |
| 9 | Figura | 0.5 | 0 | 5 | 0 | 0 | 42.0 | 0.8 | 1 | 0 | 0 | 1 | 0 |
| 10 | Garantus | 0.4 | 0 | 5 | 0 | 0 | 6.0 | 0.6 | 0 | 5 | 5 | 5 | 2.0 |
| 11 | Jenga | 0.6 | 1 | 0 | 0 | 5 | 0 | 0.4 | 0 | 0 | 0 | 0 | 0 |
| 12 | Kampana | 4.4 | 0 | 5 | 0 | 10 | 0 | 2.3 | 0 | 0 | 5 | 5 | 0 |
| 13 | Kohelia | 0.4 | 0 | 20 | 0 | 1 | 0 | 0.1 | 0 | 5 | 0 | 0 | 0 |
| 14 | Legenda | 0 | 0 | 5 | 0 | 5 | 5.0 | 0.1 | 1 | 0 | 0 | 0 | 19.0 |
| 15 | Ludwig | 0.4 | 1 | 5 | 0 | 0 | 0 | 0.2 | 0 | 1 | 0 | 0 | 0 |
| 16 | Markiza | 0.3 | 0 | 5 | 0 | 0 | 13.0 | 0.3 | 0 | 0 | 0 | 1 | 0 |
| 17 | Meteor | 0.7 | 0 | 1 | 0 | 5 | 0 | 0.4 | 0 | 0 | 0 | 5 | 0 |
| 18 | Mewa | 0.2 | 0 | 5 | 0 | 5 | 0 | 0 | 0 | 0 | 0 | 5 | 0 |
| 19 | Mulan | 0.2 | 0 | 1 | 0 | 5 | 0 | 0.1 | 0 | 0 | 5 | 5 | 0 |
| 20 | Muszelka | 3.5 | 0 | 5 | 0 | 10 | 0 | 1.6 | 0 | 0 | 5 | 5 | 0 |
| 21 | Naridana | 1.5 | 0 | 5 | 0 | 0 | 11.2 | 1.5 | 0 | 5 | 1 | 5 | 38.0 |
| 22 | Nateja | 0 | 0 | 0 | 0 | 0 | 48.0 | 0 | 0 | 0 | 0 | 0 | 56.0 |
| 23 | Ostka St. | 0.5 | 0 | 5 | 0 | 0 | 0 | 0.1 | 1 | 1 | 1 | 10 | 2.2 |
| 24 | Ostroga | 0.3 | 0 | 1 | 0 | 5 | 2.2 | 0.5 | 0 | 10 | 0 | 5 | 0 |
| 25 | Slade | 2.1 | 0 | 5 | 0 | 10 | 0 | 3.2 | 0 | 1 | 5 | 0 | 0 |
| 26 | Smuga | 1.0 | 1 | 1 | 0 | 10 | 4.2 | 0.4 | 1 | 1 | 1 | 1 | 5.0 |
| 27 | Sukces | 0.3 | 0 | 20 | 0 | 0 | 0 | 0.2 | 0 | 5 | 0 | 5 | 0 |
| 28 | Tonacja | 0.2 | 0 | 1 | 0 | 0 | 0 | 0.1 | 0 | 5 | 0 | 5 | 0 |
| 29 | Türkis | 0.7 | 1 | 5 | 0 | 5 | 0 | 1.8 | 5 | 1 | 1 | 1 | 0 |
| 30 | Zyta | 0.1 | 0 | 5 | 0 | 0 | 9 | 0.7 | 0 | 1 | 0 | 0 | 0 |
|  | Means | 0.7 | 1.0 | 4.6 | 0 | 2.5 | 5.8 | 0.7 | 0.7 | 2.1 | 2.0 | 3.2 | 4.6 |

FHBi – Fusarium head blight index, LR – leaf rust, STB – Septoria tritici blotch, TS – tan spot, SNB – Septoria nodorum blotch, YR – yellow rust
