# Supplementary material for: Performance of Winter Wheat Cultivars Grown Organically and Conventionally with Focus on Fusarium Head Blight and Fusarium Trichothecene Toxins
Source: Microorganisms. 2019 Oct 11;7(10):439. doi: 10.3390/microorganisms7100439 (PMC6843174; doi:10.3390/microorganisms7100439)
Supplement: Supplementary file 1 [file microorganisms-07-00439-s001.zip › Table S4.docx]

**Table S4.** Sowing quality parameters for of 30 winter wheat cultivars grown in conventional and organic fields

| **No.** | **Cultivar** | **Organic** | | | | | **Conventional** | | | | |
| --- | --- | --- | --- | --- | --- | --- | --- | --- | --- | --- | --- |
|  |  | **GE (%)** | **GC (%)** | **AS (%)** | **DS (%)** | **FUS (%)** | **GE (%)** | **GC (%)** | **AS (%)** | **DS (%)** | **FUS (%)** |
| 1 | Akteur | 24.0 | 79.0 | 9.0 | 6.0 | 6.0 | 96.5 | 96.5 | 1.5 | 1.5 | 0.5 |
| 2 | Alcazar | 12.5 | 84.0 | 7.0 | 8.5 | 0.5 | 92.0 | 93.5 | 2.0 | 4.0 | 0.5 |
| 3 | Anthus | 50.0 | 90.5 | 2.5 | 5.5 | 1.5 | 93.5 | 94.5 | 1.5 | 3.5 | 0.5 |
| 4 | Batuta | 52.0 | 96.0 | 0.5 | 3.5 | 0.0 | 97.5 | 98.0 | 0.0 | 1.5 | 0.5 |
| 5 | Belenus | 11.5 | 75.0 | 15.0 | 2.5 | 7.5 | 93.0 | 94.5 | 4.0 | 0.0 | 1.5 |
| 6 | Bogatka | 54.0 | 94.5 | 2.0 | 3.5 | 0.0 | 96.5 | 98.0 | 0.5 | 1.0 | 0.5 |
| 7 | Boomer | 23.5 | 89.5 | 5.5 | 3.0 | 2.0 | 97.5 | 97.5 | 1.0 | 1.0 | 0.5 |
| 8 | Dorota | 52.0 | 88.5 | 3.5 | 7.0 | 1.0 | 92.5 | 93.0 | 2.5 | 4.0 | 0.5 |
| 9 | Figura | 34.0 | 88.0 | 4.5 | 6.5 | 1.0 | 96.5 | 96.5 | 3.0 | 0.0 | 0.5 |
| 10 | Garantus | 32.0 | 87.0 | 3.5 | 8.5 | 1.0 | 94.5 | 95.0 | 2.0 | 3.0 | 0.0 |
| 11 | Jenga | 91.0 | 92.5 | 4.0 | 3.5 | 0.0 | 81.5 | 87.5 | 7.5 | 5.0 | 0.0 |
| 12 | Kampana | 79.0 | 82.5 | 10.0 | 7.5 | 0.0 | 86.5 | 91.0 | 5.0 | 4.0 | 0.0 |
| 13 | Kohelia | 83.0 | 92.0 | 1.5 | 6.5 | 0.0 | 90.5 | 97.0 | 1.0 | 1.5 | 0.5 |
| 14 | Legenda | 89.0 | 90.5 | 3.0 | 6.5 | 0.0 | 93.0 | 95.0 | 1.0 | 4.0 | 0.0 |
| 15 | Ludwig | 92.5 | 92.5 | 4.0 | 3.5 | 0.0 | 65.5 | 91.0 | 3.5 | 5.5 | 0.0 |
| 16 | Markiza | 84.0 | 95.0 | 1.5 | 0.5 | 3.0 | 91.0 | 95.5 | 2.0 | 2.0 | 0.5 |
| 17 | Meteor | 87.0 | 88.0 | 4.0 | 8.0 | 0.0 | 86.0 | 95.0 | 2.5 | 2.5 | 0.0 |
| 18 | Mewa | 93.0 | 93.5 | 1.5 | 5.0 | 0.0 | 54.0 | 88.0 | 3.0 | 7.5 | 1.5 |
| 19 | Mulan | 75.0 | 89.5 | 4.5 | 3.0 | 3.0 | 65.0 | 96.5 | 1.5 | 2.0 | 0.0 |
| 20 | Muszelka | 76.5 | 84.5 | 6.5 | 8.0 | 1.0 | 74.5 | 89.0 | 4.0 | 7.0 | 0.0 |
| 21 | Naridana | 73.5 | 90.0 | 2.0 | 8.0 | 0.0 | 86.0 | 88.5 | 4.0 | 7.0 | 0.5 |
| 22 | Nateja | 74.5 | 95.5 | 1.0 | 3.0 | 0.5 | 98.0 | 98.5 | 0.0 | 1.0 | 0.5 |
| 23 | Ostka St. | 78.0 | 90.0 | 4.5 | 5.5 | 0.0 | 95.5 | 95.5 | 1.5 | 3.0 | 0.0 |
| 24 | Ostroga | 61.0 | 84.0 | 5.0 | 6.0 | 5.0 | 75.5 | 86.5 | 4.0 | 7.5 | 2.0 |
| 25 | Slade | 58.5 | 88.0 | 5.0 | 7.0 | 0.0 | 79.5 | 83.5 | 6.5 | 7.5 | 2.5 |
| 26 | Smuga | 64.0 | 92.5 | 3.0 | 3.5 | 1.0 | 84.0 | 94.0 | 0.5 | 4.0 | 1.5 |
| 27 | Sukces | 74.5 | 91.0 | 4.5 | 4.0 | 0.5 | 83.0 | 91.0 | 3.0 | 6.0 | 0.0 |
| 28 | Tonacja | 75.0 | 91.0 | 4.0 | 4.0 | 1.0 | 93.5 | 94.5 | 2.0 | 3.0 | 0.5 |
| 29 | Türkis | 70.5 | 91.5 | 1.5 | 7.0 | 0.0 | 87.5 | 92.5 | 2.5 | 4.5 | 0.5 |
| 30 | Zyta | 71.5 | 92.5 | 2.5 | 4.5 | 0.5 | 89.0 | 94.5 | 1.0 | 4.0 | 0.5 |
|  | Mean | 63.2 | 89.3 | 4.2 | 5.3 | 1.2 | 87.0 | 93.4 | 2.5 | 3.6 | 0.6 |

GE – germination energy, GC – germination capacity, AS – abnormal seedlings, DS – dead seeds, FUS – fresh ungerminanted seeds
