# Supplementary material for: Performance of Winter Wheat Cultivars Grown Organically and Conventionally with Focus on Fusarium Head Blight and Fusarium Trichothecene Toxins
Source: Microorganisms. 2019 Oct 11;7(10):439. doi: 10.3390/microorganisms7100439 (PMC6843174; doi:10.3390/microorganisms7100439)
Supplement: Supplementary file 1 [file microorganisms-07-00439-s001.zip › Table S5.docx]

**Table S5.** Concentrations of ergosterol (mg kg^-1^) and type B trichothecenes (μg kg^-1^) in grain of 30 winter wheat cultivars grown in conventional and organic fields

| **No.** | **Cultivar** | **Conventional** | | | | | | | **Organic** | | | | | | |
| --- | --- | --- | --- | --- | --- | --- | --- | --- | --- | --- | --- | --- | --- | --- | --- |
|  |  | **ERG** | **DON** | **FUS-X** | **3-Ac DON** | **15-Ac DON** | **NIV** | **TCT B** | **ERG** | **DON** | **FUS-X** | **3-Ac DON** | **15-Ac DON** | **NIV** | **TCT B** |
| 1 | Akteur | 0.85 | 45.2 | 5.6 | 6.4 | 0.8 | 0 | 57.8 | 3.46 | 49.3 | 0.2 | 2.6 | 0.4 | 8.7 | 61.2 |
| 2 | Alcazar | 0.34 | 103.6 | 0 | 12.7 | 0.9 | 11.5 | 128.6 | 2.48 | 348.3 | 0.7 | 6.2 | 1.7 | 27.5 | 384.5 |
| 3 | Anthus | 0.68 | 444.8 | 0 | 2.3 | 2.5 | 10.6 | 460.2 | 1.78 | 222.7 | 0 | 4.8 | 0.4 | 16.7 | 244.6 |
| 4 | Batuta | 1.25 | 45.3 | 0 | 16.0 | 0 | 12.6 | 73.9 | 2.43 | 30.1 | 0.4 | 4.0 | 0.2 | 5.3 | 39.9 |
| 5 | Belenus | 0.63 | 122.4 | 0 | 2.4 | 2.1 | 0 | 126.9 | 0.68 | 14.8 | 0 | 3.0 | 0.4 | 3.2 | 21.5 |
| 6 | Bogatka | 0.55 | 24.2 | 0.8 | 7.9 | 0 | 7.4 | 40.2 | 1.39 | 57.5 | 0 | 3.6 | 0.5 | 10.0 | 71.6 |
| 7 | Boomer | 0.28 | 35.6 | 0 | 6.2 | 0 | 5.4 | 47.2 | 0.26 | 20.7 | 0.2 | 3.2 | 0.3 | 2.8 | 27.2 |
| 8 | Dorota | 0.48 | 70.5 | 0 | 6.4 | 2.1 | 4.0 | 82.9 | 2.43 | 33.4 | 0.6 | 3.1 | 0 | 0 | 37.1 |
| 9 | Figura | 0.83 | 38.0 | 0 | 10.9 | 0.3 | 4.0 | 53.3 | 2.64 | 3.0 | 0 | 2.4 | 0.3 | 4.4 | 10.1 |
| 10 | Garantus | 1.24 | 258.9 | 0 | 30.3 | 2.9 | 16.8 | 308.9 | 0.63 | 52.3 | 0.4 | 3.6 | 0.4 | 8.7 | 65.4 |
| 11 | Jenga | 0.58 | 140.6 | 0 | 7.9 | 2.7 | 6.3 | 157.6 | 1.48 | 94.5 | 0.7 | 5.3 | 0.3 | 5.0 | 105.8 |
| 12 | Kampana | 0.83 | 87.7 | 0 | 8.8 | 0.9 | 6.1 | 103.5 | 0.59 | 249.4 | 2.7 | 2.4 | 1.1 | 22.6 | 278.1 |
| 13 | Kohelia | 0.69 | 6.4 | 0 | 2.4 | 0.6 | 8.1 | 17.5 | 1.25 | 86.2 | 0.2 | 3.5 | 0.3 | 10.0 | 100.2 |
| 14 | Legenda | 1.14 | 11.0 | 0.6 | 4.9 | 0.3 | 1.7 | 18.5 | 1.49 | 97.6 | 0 | 4.8 | 0.4 | 5.3 | 108.1 |
| 15 | Ludwig | 0.37 | 43.4 | 0.7 | 5.5 | 0.3 | 2.2 | 52.1 | 2.17 | 49.4 | 0 | 3.2 | 0.6 | 6.0 | 59.1 |
| 16 | Markiza | 0.72 | 5.8 | 0 | 2.4 | 0.7 | 0 | 8.9 | 0.59 | 62.1 | 0.3 | 6.0 | 0.2 | 6.2 | 74.8 |
| 17 | Meteor | 1.44 | 16.5 | 0.6 | 6.7 | 0.4 | 1.6 | 25.8 | 1.39 | 77.9 | 0.5 | 4.5 | 0 | 9.2 | 92.2 |
| 18 | Mewa | 0.59 | 59.7 | 0.8 | 6.2 | 0.4 | 1.2 | 68.3 | 0.38 | 68.0 | 0.5 | 4.3 | 0.4 | 3.4 | 76.6 |
| 19 | Mulan | 1.85 | 20.4 | 0 | 4.7 | 0.3 | 1.6 | 26.9 | 1.53 | 7.7 | 0.3 | 2.4 | 0.2 | 1.5 | 12.1 |
| 20 | Muszelka | 0.68 | 151.1 | 0.4 | 4.7 | 0.5 | 4.9 | 161.6 | 1.94 | 222.6 | 0.3 | 4.3 | 1.0 | 29.5 | 257.6 |
| 21 | Naridana | 0.38 | 70.9 | 11.6 | 18.5 | 14.3 | 18.9 | 134.3 | 0.29 | 9.9 | 2.9 | 2.3 | 3.3 | 4.2 | 22.6 |
| 22 | Nateja | 0.52 | 18.7 | 4.0 | 8.8 | 3.4 | 10.7 | 45.5 | 0.33 | 2.3 | 2.4 | 2.0 | 2.9 | 3.3 | 13.0 |
| 23 | Ostka St. | 0.26 | 106.9 | 0.9 | 2.4 | 2.3 | 2.3 | 114.8 | 2.49 | 3.7 | 2.2 | 0 | 2.8 | 2.9 | 11.6 |
| 24 | Ostroga | 0.46 | 303.1 | 0 | 5.6 | 2.0 | 10.4 | 321.1 | 0.27 | 9.9 | 2.3 | 0 | 2.8 | 3.2 | 18.2 |
| 25 | Slade | 0.73 | 117.1 | 0 | 8.0 | 1.2 | 3.1 | 129.3 | 1.69 | 8.7 | 0 | 2.0 | 0 | 3.7 | 14.3 |
| 26 | Smuga | 0.69 | 53.3 | 0 | 2.4 | 0.3 | 0 | 55.9 | 0.93 | 7.8 | 2.2 | 1.9 | 2.7 | 3.0 | 17.5 |
| 27 | Sukces | 0.46 | 48.0 | 0 | 2.4 | 1.0 | 7.4 | 58.7 | 0.49 | 3.7 | 2.2 | 2.0 | 2.7 | 4.8 | 15.3 |
| 28 | Tonacja | 1.53 | 34.5 | 0 | 8.0 | 0 | 0 | 42.5 | 1.49 | 8.0 | 0 | 1.9 | 2.6 | 3.9 | 16.5 |
| 29 | Türkis | 0.59 | 39.7 | 0 | 2.3 | 0.8 | 3.4 | 46.2 | 2.46 | 6.3 | 2.1 | 1.8 | 2.6 | 3.4 | 16.3 |
| 30 | Zyta | 0.47 | 19.9 | 0 | 4.3 | 0 | 6.6 | 30.8 | 1.20 | 2.1 | 2.2 | 1.9 | 2.7 | 3.5 | 12.6 |
|  | Means | 0.74 | 84.8 | 0.9 | 7.3 | 1.5 | 5.6 | 100.0 | 1.42 | 63.7 | 0.9 | 3.1 | 1.1 | 7.4 | 76.2 |

ERG – ergosterol, DON – deoxynivalenol, FUS-X – fusarenon X, 3-AcDON – 3-acetyl deoxynivalenol, 15-AcDON - 15-acetyl deoxynivalenol, NIV – nivalenol, TCT B – sum of type B trichothecenes
