# Supplementary material for: Performance of Winter Wheat Cultivars Grown Organically and Conventionally with Focus on Fusarium Head Blight and Fusarium Trichothecene Toxins
Source: Microorganisms. 2019 Oct 11;7(10):439. doi: 10.3390/microorganisms7100439 (PMC6843174; doi:10.3390/microorganisms7100439)
Supplement: Supplementary file 1 [file microorganisms-07-00439-s001.zip › Table S6.docx]

**Table S6**. Concentrations of type A trichothecenes (μg kg^-1^) in grain of 30 winter wheat cultivars grown in conventional and organic fields

| **No.** | **Cultivar** | **Conventional** | | | | | | | **Organic** | | | | | | |
| --- | --- | --- | --- | --- | --- | --- | --- | --- | --- | --- | --- | --- | --- | --- | --- |
|  |  | **STO** | **T-2 Tetraol** | **T-2 Triol** | **DAS** | **HT-2** | **T-2** | **TCT A** | **STO** | **T-2 Tetraol** | **T-2 Triol** | **DAS** | **HT-2** | **T-2** | **TCT A** |
| 1 | Akteur | 5.0 | 0.9 | 0.5 | 2.4 | 1.9 | 0.9 | 11.6 | 1.0 | 0.1 | 0.1 | 0.4 | 0.2 | 0 | 1.8 |
| 2 | Alcazar | 4.1 | 1.0 | 0.2 | 2.0 | 0.8 | 0.1 | 8.2 | 6.4 | 0.1 | 0.1 | 0.9 | 0.3 | 0 | 7.8 |
| 3 | Anthus | 4.4 | 1.4 | 0.2 | 0.1 | 2.3 | 0 | 8.3 | 4.0 | 0.0 | 0 | 0.0 | 0.2 | 0 | 4.4 |
| 4 | Batuta | 0.5 | 0.2 | 0.1 | 0.1 | 0.1 | 0 | 1.0 | 1.5 | 0.0 | 0.1 | 0.5 | 0.1 | 0 | 2.3 |
| 5 | Belenus | 0.6 | 0.2 | 0.1 | 0.3 | 0.3 | 0.1 | 1.7 | 2.1 | 0.0 | 0.1 | 0.0 | 0.3 | 0 | 2.5 |
| 6 | Bogatka | 2.2 | 0.8 | 0.2 | 0.3 | 1.3 | 0 | 4.8 | 3.3 | 0.5 | 0 | 0.1 | 0.2 | 0 | 4.1 |
| 7 | Boomer | 0.2 | 0.1 | 0.0 | 0.2 | 0.2 | 0 | 0.7 | 8.9 | 0.1 | 0 | 0.4 | 0.2 | 0 | 9.6 |
| 8 | Dorota | 2.4 | 0.4 | 0.1 | 0.1 | 0.3 | 0 | 3.3 | 1.6 | 0.1 | 0.0 | 0.1 | 0.3 | 0 | 2.1 |
| 9 | Figura | 1.1 | 0.2 | 0.1 | 0.1 | 0.3 | 0.1 | 1.8 | 0.5 | 0.0 | 0 | 0.4 | 0.0 | 0 | 1.0 |
| 10 | Garantus | 2.3 | 5.6 | 1.9 | 0.2 | 20.5 | 0 | 30.5 | 1.1 | 0.0 | 0 | 0.8 | 0.2 | 0 | 2.1 |
| 11 | Jenga | 1.7 | 0.4 | 0.1 | 0.7 | 0.2 | 0 | 3.1 | 3.8 | 0.4 | 0.1 | 0.2 | 0.3 | 0.4 | 5.2 |
| 12 | Kampana | 1.3 | 0.4 | 0.1 | 0.1 | 0.4 | 0 | 2.3 | 12.0 | 1.1 | 0.1 | 0.1 | 0.2 | 0.2 | 13.8 |
| 13 | Kohelia | 2.1 | 0.2 | 0.1 | 0.8 | 0.3 | 0 | 3.5 | 4.4 | 0.3 | 0.1 | 0.1 | 0.3 | 0.1 | 5.4 |
| 14 | Legenda | 1.1 | 0.4 | 0.1 | 0.2 | 0.1 | 0.1 | 2.0 | 1.7 | 0.3 | 0.1 | 0.1 | 0.2 | 0 | 2.5 |
| 15 | Ludwig | 1.8 | 0.3 | 0.1 | 2.0 | 0.4 | 0.1 | 4.7 | 2.1 | 0.2 | 0.1 | 0.1 | 0.4 | 0.1 | 3.1 |
| 16 | Markiza | 0.9 | 0.3 | 0.2 | 1.0 | 0.4 | 0 | 2.8 | 2.2 | 0.1 | 0.1 | 1.1 | 0.2 | 0 | 3.7 |
| 17 | Meteor | 0.7 | 0.3 | 0.1 | 2.3 | 0.4 | 0 | 3.8 | 1.1 | 0.3 | 0.2 | 0.1 | 0.3 | 0 | 2.0 |
| 18 | Mewa | 2.1 | 0.2 | 0 | 2.7 | 0.4 | 0.1 | 5.5 | 1.2 | 0.2 | 0 | 0.0 | 0.2 | 0 | 1.6 |
| 19 | Mulan | 1.8 | 0.1 | 0.2 | 1.6 | 0.2 | 0 | 4.0 | 2.0 | 0.3 | 0.1 | 0.7 | 0.2 | 0 | 3.3 |
| 20 | Muszelka | 5.5 | 0.9 | 0.2 | 2.1 | 0.6 | 0 | 9.4 | 4.3 | 0.4 | 0 | 0.1 | 0.4 | 0 | 5.3 |
| 21 | Naridana | 4.7 | 0.5 | 0 | 0.1 | 0.2 | 0 | 5.5 | 5.0 | 0.7 | 0.2 | 0.5 | 0.3 | 0 | 6.7 |
| 22 | Nateja | 1.0 | 0.3 | 0 | 0.1 | 0.1 | 0 | 1.5 | 0.8 | 0.1 | 0.1 | 0.1 | 0.2 | 0 | 1.2 |
| 23 | Ostka St. | 6.8 | 0.0 | 0.0 | 0.5 | 0.2 | 0 | 7.5 | 10.0 | 1.0 | 0.2 | 0.5 | 0.5 | 0 | 12.2 |
| 24 | Ostroga | 16.9 | 0.8 | 0 | 0.0 | 0.1 | 0 | 17.9 | 2.6 | 0.3 | 0.1 | 0.1 | 0.2 | 0 | 3.4 |
| 25 | Slade | 1.7 | 0.5 | 0.1 | 0.1 | 0.7 | 0 | 3.1 | 4.6 | 0.4 | 0.1 | 0.1 | 0.3 | 0 | 5.4 |
| 26 | Smuga | 5.6 | 0.3 | 0 | 0.6 | 0.2 | 0 | 6.6 | 8.8 | 0.4 | 0.2 | 0.1 | 0.3 | 0 | 9.8 |
| 27 | Sukces | 0 | 0.1 | 0.3 | 0.1 | 0.7 | 0 | 1.1 | 4.9 | 0.2 | 0.3 | 0.1 | 0.4 | 0 | 5.9 |
| 28 | Tonacja | 1.9 | 0.1 | 0.1 | 0.4 | 0.2 | 0 | 2.8 | 2.5 | 0.4 | 0.1 | 0.1 | 0.4 | 0 | 3.4 |
| 29 | Türkis | 1.1 | 0.2 | 0.1 | 0.7 | 0.2 | 0 | 2.2 | 5.5 | 0.6 | 0.1 | 0.0 | 0.8 | 0 | 7.1 |
| 30 | Zyta | 1.4 | 0.0 | 0.1 | 0.0 | 1.2 | 0 | 2.8 | 8.3 | 3.2 | 0.3 | 0.4 | 2.3 | 0 | 14.5 |
|  | Means | 2.8 | 0.6 | 0.2 | 0.7 | 1.2 | 0.1 | 5.5 | 3.9 | 0.4 | 0.1 | 0.3 | 0.3 | 0.0 | 5.1 |

STO – scirpentriol, DAS – diacetoxyscirpenol, HT-2 - HT-2 toxin, T-2 – T-2 toxin, TCT A - sum of type A trichothecenes
