# Supplementary material for: Performance of Winter Wheat Cultivars Grown Organically and Conventionally with Focus on Fusarium Head Blight and Fusarium Trichothecene Toxins
Source: Microorganisms. 2019 Oct 11;7(10):439. doi: 10.3390/microorganisms7100439 (PMC6843174; doi:10.3390/microorganisms7100439)
Supplement: Supplementary file 1 [file microorganisms-07-00439-s001.zip › Table S7.docx]

**Table S7.** Concentration of DNA (pg 100ng^-1^ of wheat DNA) of five *Fusarium* species in grain of 30 winter wheat cultivars grown in conventional and organic fields

| **No.** | **Cultivar** | **Conventional** | | | | | | **Organic** | | | | | |
| --- | --- | --- | --- | --- | --- | --- | --- | --- | --- | --- | --- | --- | --- |
|  |  | ***Fa* DNA** | ***Fc* DNA** | ***Fg* DNA** | ***Fp* DNA** | ***Fsp* DNA** | ***Fusarium* DNA** | ***Fa* DNA** | ***Fc* DNA** | ***Fg* DNA** | ***Fp* DNA** | ***Fsp* DNA** | ***Fusarium* DNA** |
| 1 | Akteur | 5.6 | 13.3 | 16.5 | 56.5 | 0.4 | 92.3 | 24.1 | 21.9 | 50.1 | 105.0 | 13.4 | 214.4 |
| 2 | Alcazar | 15.3 | 21.1 | 44.9 | 132.4 | 36.6 | 250.3 | 27.3 | 31.7 | 195.1 | 222.0 | 279.3 | 755.4 |
| 3 | Anthus | 0.9 | 11.1 | 17.9 | 20.2 | 3.6 | 53.6 | 1.8 | 19.8 | 3.3 | 68.4 | 5.7 | 99.0 |
| 4 | Batuta | 4.5 | 0.0 | 1.3 | 9.4 | 0.2 | 15.4 | 0.4 | 0.0 | 0.5 | 14.1 | 0.3 | 15.3 |
| 5 | Belenus | 13.2 | 12.5 | 51.0 | 36.2 | 2.7 | 115.5 | 9.6 | 35.6 | 55.7 | 182.1 | 60.5 | 343.5 |
| 6 | Bogatka | 0.0 | 0.0 | 1.3 | 18.8 | 0.0 | 20.1 | 0.4 | 415.5 | 39.5 | 118.2 | 0.4 | 574.0 |
| 7 | Boomer | 6.6 | 0.0 | 6.1 | 57.5 | 11.8 | 82.1 | 3.1 | 25.0 | 15.7 | 109.3 | 16.8 | 170.0 |
| 8 | Dorota | 16.4 | 0.0 | 10.8 | 18.1 | 6.6 | 52.0 | 13.4 | 0.0 | 58.5 | 38.5 | 3.6 | 114.0 |
| 9 | Figura | 2.9 | 346.2 | 17.1 | 38.8 | 0.3 | 405.2 | 25.1 | 0.0 | 83.3 | 72.2 | 17.2 | 197.8 |
| 10 | Garantus | 1.4 | 0.0 | 32.6 | 42.2 | 11.1 | 87.3 | 5.2 | 0.0 | 85.4 | 220.8 | 1.8 | 313.1 |
| 11 | Jenga | 12.4 | 32.1 | 23.8 | 24.4 | 25.0 | 117.6 | 54.3 | 23.2 | 96.4 | 73.2 | 2.5 | 249.5 |
| 12 | Kampana | 106.7 | 0.0 | 50.6 | 61.3 | 113.3 | 331.8 | 49.0 | 0.0 | 92.3 | 168.0 | 67.4 | 376.7 |
| 13 | Kohelia | 0.2 | 10.0 | 4.0 | 19.0 | 0.2 | 33.4 | 3.3 | 13.3 | 34.3 | 51.5 | 0.0 | 102.4 |
| 14 | Legenda | 18.8 | 38.5 | 15.0 | 15.9 | 5.7 | 93.9 | 2.2 | 44.1 | 57.6 | 43.7 | 9.3 | 156.8 |
| 15 | Ludwig | 1.2 | 25.8 | 18.1 | 15.6 | 3.4 | 64.1 | 31.4 | 15.4 | 33.2 | 80.8 | 0.0 | 160.7 |
| 16 | Markiza | 17.4 | 0.0 | 8.0 | 43.2 | 107.3 | 175.8 | 3.5 | 93.3 | 14.8 | 63.9 | 3.9 | 179.5 |
| 17 | Meteor | 30.5 | 0.0 | 14.6 | 42.2 | 2.0 | 89.2 | 109.0 | 28.2 | 13.8 | 139.4 | 271.8 | 562.3 |
| 18 | Mewa | 0.0 | 10.5 | 5.4 | 12.7 | 0.0 | 28.6 | 1.7 | 18.2 | 89.0 | 61.5 | 0.5 | 170.9 |
| 19 | Mulan | 0.6 | 0.0 | 23.4 | 15.1 | 1.1 | 40.3 | 24.6 | 0.0 | 29.6 | 155.4 | 5.2 | 214.8 |
| 20 | Muszelka | 8.4 | 0.0 | 40.3 | 28.2 | 9.9 | 86.9 | 55.9 | 54.8 | 14.6 | 81.9 | 160.0 | 367.1 |
| 21 | Naridana | 5.1 | 74.5 | 46.4 | 22.8 | 1.0 | 149.8 | 1.9 | 14.6 | 130.6 | 53.9 | 0.3 | 201.3 |
| 22 | Nateja | 0.7 | 18.2 | 0.7 | 21.4 | 2.4 | 43.3 | 0.2 | 0.0 | 0.9 | 24.8 | 1.2 | 27.1 |
| 23 | Ostka St. | 18.3 | 35.8 | 79.5 | 45.1 | 13.5 | 192.2 | 5.0 | 12.5 | 30.6 | 41.9 | 3.0 | 93.0 |
| 24 | Ostroga | 5.8 | 10.5 | 33.6 | 35.7 | 0.0 | 85.6 | 184.6 | 0.0 | 147.5 | 130.2 | 73.8 | 536.2 |
| 25 | Slade | 13.1 | 10.0 | 76.0 | 71.5 | 0.2 | 170.8 | 150.3 | 245.0 | 280.0 | 180.5 | 350.0 | 1205.8 |
| 26 | Smuga | 3.9 | 18.4 | 24.7 | 14.4 | 0.2 | 61.6 | 1.9 | 27.3 | 118.8 | 31.8 | 4.7 | 184.6 |
| 27 | Sukces | 9.0 | 0.0 | 6.5 | 50.4 | 60.9 | 126.8 | 35.2 | 0.0 | 68.5 | 88.8 | 18.0 | 210.4 |
| 28 | Tonacja | 3.4 | 0.0 | 1.8 | 15.5 | 0.0 | 20.7 | 0.5 | 18.2 | 30.3 | 122.7 | 5.2 | 176.9 |
| 29 | Türkis | 1.6 | 11.8 | 9.2 | 38.1 | 7.9 | 68.5 | 52.8 | 0.0 | 124.4 | 106.6 | 139.0 | 422.8 |
| 30 | Zyta | 0.0 | 0.0 | 1.0 | 18.8 | 24.3 | 44.2 | 28.7 | 34.9 | 16.6 | 96.0 | 0.5 | 176.8 |
|  | Means | 10.8 | 23.3 | 22.7 | 34.7 | 15.1 | 106.6 | 30.2 | 39.7 | 67.0 | 98.2 | 50.5 | 285.7 |

*Fa* – *F. avenaceum*, *Fc* – *F. culmorum*, *Fg* – *F. graminearum*, *Fp* – *F. poae*, *Fsp* – *F. sporotrichioides*, *Fusarium* DNA – total DNA of five *Fusarium* species
